# Staged open reduction and internal fixation with double-locking plates to treat bilateral distal femur periprosthetic fractures after total knee arthroplasty: A case report

**DOI:** 10.3389/fsurg.2022.987953

**Published:** 2023-01-06

**Authors:** Jiangpeng Wu, Zheng Li, Jiang Huang, Xufeng Jiao, Guanglei Cao

**Affiliations:** Department of Orthopedic Surgery, Xuanwu Hospital, Capital Medical University, Beijing, China

**Keywords:** case report, total knee arthroplasty, distal femur periprosthetic fracture, double-locking plate, delayed union

## Abstract

**Background:**

The incidence of periprosthetic fractures after total knee arthroplasty (TKA) increases in parallel with the number of procedures. Comminuted fractures along the primary fracture line extending to the edge of the prosthesis are challenging, and bilateral fractures are rarely reported, especially with open injuries.

**Case presentation:**

A 65-year-old female had undergone bilateral TKA in our hospital 5 years before admission. She was admitted with a traumatic bilateral Rorabeck type II B distal femur periprosthetic fracture (closed right, open left, Gustilo II) and was treated with bilateral staged open reduction and internal fixation (ORIF) with double-locking plates. The patient experienced a prolonged delayed fracture union and finally healed around 21 months postoperatively. The function was satisfactory after 4 years of follow-up.

**Conclusion:**

ORIF with double-locking plates can be used to treat Rorabeck II B periprosthetic fracture where the primary fracture line extends beyond the edge of the prosthesis; however, there may be delayed healing or nonunion. Patients need to undergo long-term rehabilitation and endure long disability times and require good rehabilitation nursing care. Once they achieve bone healing, the treatment achieves bone preservation and substantial prosthesis survival.

## Background

Total knee arthroplasty (TKA) is an effective treatment for older patients with end-stage knee osteoarthritis. The incidence of periprosthetic fractures after TKA increases in parallel with the number of procedures. Comminuted fractures along the primary fracture line extending to the edge of the prosthesis are challenging, and bilateral fractures are rarely reported, especially with open injuries. We report a case of bilateral Rorabeck II B periprosthetic fractures treated with staged bilateral open reduction and internal fixation (ORIF) with double-locking plates 5 years after bilateral TKA. Fracture healing was achieved despite a lengthy postoperative recovery process, and the functional result was satisfactory.

## Case presentation

**Figure 1:**
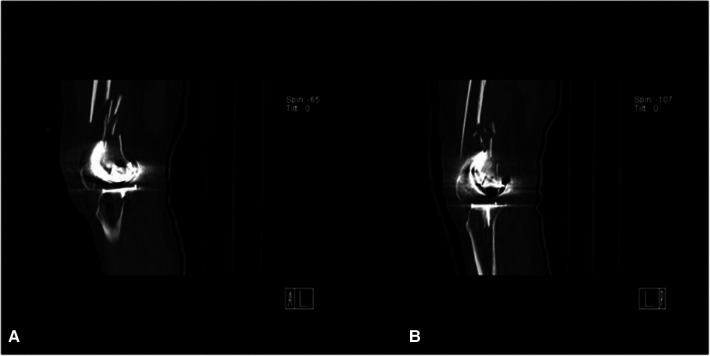
Preoperative sagittal CT of bilateral knees: the right knee (**A**), the left knee (**B**).

**Figure 2:**
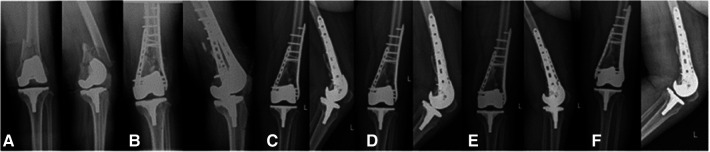
Anteroposterior and lateral radiographs of the left knee: preoperative x-ray image (**A**), postoperative x-ray images (**B**), radiograph 7 months after surgery (**C**), radiograph 9 months after surgery (**D**), radiograph 21 months after surgery (**E**), radiograph 34 months after surgery (**F**).

**Figure 3:**
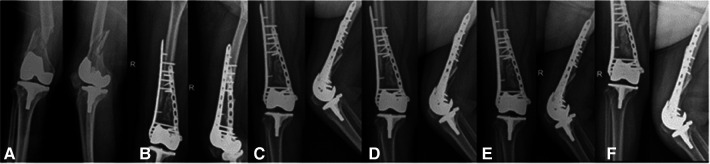
Anteroposterior and lateral radiographs of the right knee: preoperative x-ray image (**A**), postoperative x-ray images (**B**), radiograph 7 months after surgery (**C**), radiograph 9 months after surgery (**D**), radiograph 21 months after surgery (**E**), radiograph 34 months after surgery (**F**).

**Figure 4:**
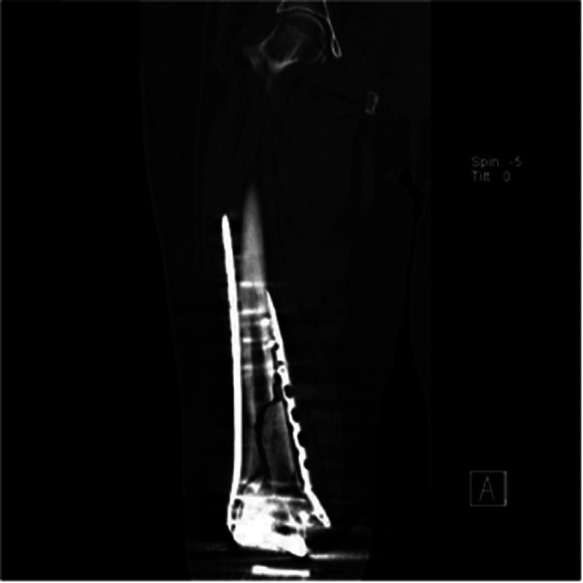
Coronal CT image of the right femur one year after surgery.

A 65-year-old female was admitted to our hospital with swelling, pain, and deformity of both knee joints caused by a car accident. The patient had undergone successful bilateral TKA at the hospital 5 years before admission and had an uneventful postoperative course. On admission, her physical examination was notable for swelling and deformity at the distal end of both thighs. An open wound of 3 cm on the left lateral thigh was accompanied by active bleeding. There was local tenderness, palpable bone friction, abnormal movement, and normal sensation and movement at the distal end of both lower limbs. Radiographs revealed a comminuted fracture of both distal femurs, with the fracture line either close to or partially distal to the anterior flange edge of the femoral prosthesis, especially on the left side (Figures 1, 2A, 3A). The clinical impression was distal femur periprosthetic fracture (DFPF; bilateral, Rorabeck II B) and distal femur open fracture (left, Gustilo II) after TKA. Debridement and suture of the left side was performed in the emergency department 6 h after the injury and was admitted to the hospital for further treatment. Then distal tibial traction stabilized the fracture, Intravenous infusion of piperacillin-sulbactam for 2 weeks to prevent infection. After the examination, the right fracture was first treated with ORIF with double-locking plates and iliac bone grafting. Two weeks later, after the left wound had healed, the same operation was performed. We adopted the original incision approach and extended it proximally to expose the fracture site. The degree of fracture comminution was severe, a massive free bone fragment was found in the fracture site, and the right side was more severe than the left. Although the fracture line had extended the anterior flange edge of the femoral prosthesis, both prostheses were stable (Figures 4). The fixations were stable despite the shortage of distal bone stock. The range of motion was 90°–0° intraoperatively, and the fracture alignment was satisfactory postoperatively (Figures 2B, 3B).

**Figure 5:**
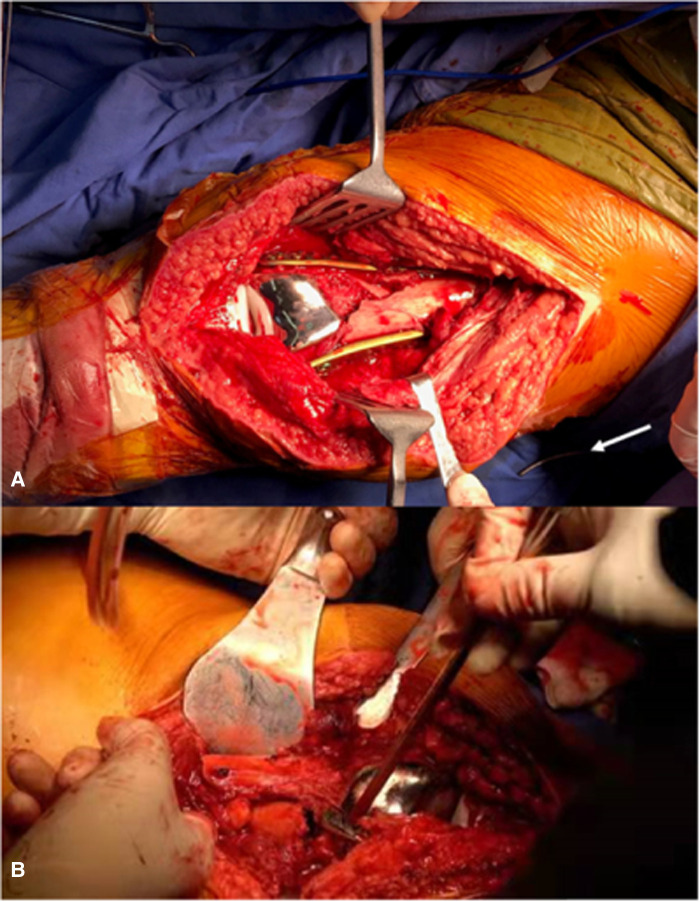
Fracture end (arrows), intraoperative image of the left knee (**A**), intraoperative image of the right knee (**B**).

Postoperative bilateral wounds healed uneventfully. We did not apply a brace. Within 6 months postoperatively, the range of knee flexion was increased using functional exercise in bed. Monthly x-rays were taken to monitor fracture healing, and teriparatide was given subcutaneously for 1 year. At 7 months postoperatively (Figures 2C, 3C), shockwave therapy was regularly performed for 16 months due to poor callus growth at the fractured sites of bilateral fractures (more evident on the right side). At 9 months postoperatively, callus formation began on the left side (Figure 2D); however, healing on the right side was not evident (Figure 3D). Computed tomography revealed no apparent callus formation between the bone fragments one year after surgery (Figure 5). The patient was hospitalized again for autologous marrow injection at the fracture site. We performed a puncture from the posterior superior iliac spine, extracted about 40 ml of bone marrow blood, and then injected it into the nonunion site of the fracture under fluoroscopy. The left lower limb was partially weight-bearing under the protection of double crutches, while the right lower limb remained non-weight-bearing. An x-ray revealed callus formation on the right side 21 months after surgery (Figure 3E), indicating partial weight-bearing. At 34 months postoperatively, films revealed healing (Figure 3F), and the patient could walk normally without the walker. At 47 months postoperatively, the range of motion on the left was 75–5–0°; on the right, it was 80–5–0°. The hospital of special surgery (HSS) score was 70 on the left and 76 on the right.

## Discussion

The management of DFPF after TKA presents challenges and carries an incidence of complications between 0.3% and 2.5% (1–4). Rorabeck et al. proposed the corresponding classification: Type I, the fracture is without apparent displacement; Type II, fracture displacement more than 5 mm, and angle more than 5°, with stable prosthesis (4). These types were subdivided into type II A (non-comminuted fracture) and type II B (comminuted fracture). Type III represents prosthesis loosening regardless of fracture site displacement. Although this classification is widely used, it has limited value for guiding clinical decision-making, especially for some type II fractures. Types should be subdivided according to the location of the fracture concerning the anterior flange of the femoral prosthesis. In some cases of type II fractures, the primary fracture line is near or distal to the proximal anterior flange of the femoral prosthesis; in these cases, treatment options and outcomes may differ entirely.

Type II B fractures are often combined with bone defects. When the fracture line is located at the edge of the prosthesis or beyond the anterior flange of the femoral prosthesis, there is a shortage of bone stock of the distal fragment for adequate fixation; this condition affects the stability of internal fixation and leads to fixation failure. Herrera et al. reported that such fractures had high rates of complications, with a nonunion rate of 9%, a fixation failure rate of 4%, an infection rate of 3%, and a revision surgery rate of 13% (5). The management of DFPF after TKA is challenging, especially for some type II fractures. To the best of our knowledge, there are a few reports of bilateral simultaneous femoral periprosthetic fractures after TKA, and only one case of concurrent open injuries (6), making treatment more complex, challenging, and uncertain.

A stable, undisplaced fracture can be treated conservatively. Satisfactory intramedullary and extramedullary fixation can be achieved for stable prostheses with fracture lines more than 2 cm proximal to the prosthesis. Distal femur replacement is appropriate for fractures with loosened prostheses. However, there is controversy regarding the treatment of fractures located at the edge of the anterior flange of the femoral prosthesis or even partial fractures beyond the edge with a stable prosthesis.

Ristevski et al. analyzed 719 cases of DFPF after TKA and found that surgery was superior to non-surgical treatment while locking plates had a lower malunion rate than retrograde intramedullary nail; however, the nonunion rate also increased (7). The use of bilateral double plates can increase the angulation stability of the fracture site, thus reducing the incidence of internal fixation failure. However, double-locking plates treatment damage the blood supply of the fracture end, resulting in reduced blood supply and increased nonunion rate. Distal femoral replacement (DFR) may lead to faster postoperative recovery (8). However, due to the limited fixation strength between the prosthesis and femur, the long-term survival rate of the prosthesis is low (8), which increases the difficulty of subsequent revisions and is not suitable for young and active patients. Vertesich et al. followed 30 patients after DFR surgery and found that the survival rate without revision was 74.8% one year after surgery, 62.5% three years after surgery, and 40.9% 10 years after surgery (9). These fractures can be treated with locked-plate internal fixation with predictable results. Wadhwa et al. reported on 1,484 patients in 58 studies and found no differences in complications between DFR and ORIF around DFPF (10); the ORIF group had a greater range of motion of the knee; however, there was no difference in functional outcomes.

In the present case, due to the severe comminution of the fracture site and the fracture line close to the prosthesis, there was insufficient good-quality bone available for fixation of the distal fracture fragment, and internal fixation was extremely difficult. Although distal femur replacement is more straightforward and allows patients to rehabilitate earlier, the rate of postoperative prosthesis loosening and the difficulty of subsequent revision is significantly increased because the patient is relatively young and active. In addition, because a posterior-stabilizing (PS) prosthesis was used for the first time, the prosthesis occupied the femoral intercondyle; there remained the problem of insufficient bone stock at the distal fragment to fix the retrograde intramedullary nail. Therefore, we used ORIF with double-locking plates. The original extended incision can be used for more convenient exposure and fixation. The most significant disadvantages of this method are disruption of local blood supply and higher probability of delayed or nonunion fracture; this patient also experienced a long fracture healing time.

One of the most critical factors governing the successful healing of this patient without plate and screw loosening or fracture was the patient's good compliance with rehabilitation. The patient could endure a long period of disability before fracture healing, and this feat is difficult for a typical individual. Therefore, for such cases, if ORIF with double-locking plates is planned, it is necessary to maintain good communication with the patient preoperatively, improve the patient's compliance, and ensure close follow-up and proper rehabilitation guidance postoperatively.

To promote fracture union, teriparatide injection, shock wave, and autologous bone marrow injection were used. Although it can't be conclusively confirmed which approach is more critical, these combined measures are a reasonable approach for this type of fracture. We consider that delayed weight bearing is an important cause of fracture union. Adherence to free weight bearing before internal fixation failure plays an important role in successful fracture union.

## Conclusion

We reported an older individual who suffered a bilateral Rorabeck II B periprosthetic fracture in which the fracture line was very close to the femoral component; she was treated with staged bilateral ORIF with double-locking plates. In similar patients, bilateral locking plates can provide sufficient stability to the fracture site. Once the fracture heals, patients can achieve maximum preservation of the native bone stock. Preoperatively, we need to communicate with the patient and family members to improve postoperative compliance and prepare the family to cope with a long period of disability. We should follow these patients closely and adjust the rehabilitation process according to fracture healing to avoid fixation failure caused by delayed healing or nonunion.

## Data Availability

The original contributions presented in the study are included in the article/Supplementary Material, further inquiries can be directed to the corresponding author/s.

## References

[1] Delport PH Van Audekercke R Martens M Mulier JC. Conservative treatment of ipsilateral supracondylar femoral fracture after total knee arthroplasty. J Trauma. (1984) 24(9):846–9. 10.1097/00005373-198409000-00013 6481837

[2] Culp RW Schmidt RG Hanks G Mak A Esterhai JL Heppenstall RB. Supracondylar fracture of the femur following prosthetic knee arthroplasty. Clin Orthop. (1987) 222:212–22. 3621724

[3] Figgie MP Goldberg VM Figgie HE Sobel M. The results of treatment of supracondylar fracture above total knee arthroplasty. J Arthroplasty. (1990) 5(3):267–76. 10.1016/S0883-5403(08)80082-4 2230824

[4] Rorabeck CH Taylor JW. Classification of periprosthetic fractures complicating total knee arthroplasty. Orthop Clin North Am. (1999) 30(2):209–14. 10.1016/S0030-5898(05)70075-4 10196422

[5] Herrera DA Kregor PJ Cole PA Levy BA Jonsson A Zlowodzki M. Treatment of acute distal femur fractures above a total knee arthroplasty: systematic review of 415 cases (1981–2006). Acta Orthop. (2008) 79(1):22–7. 10.1080/17453670710014716 18283568

[6] Neal DC Sambhariya V Rahman SK Tran A Wagner RA. Single stage bilateral flexible intramedullary fixation of periprosthetic distal femur fractures. Arthroplast Today. (2019) 5(4):421–6. 10.1016/j.artd.2019.08.001 31886383 PMC6920723

[7] Ristevski B Nauth A Williams DS Hall JA Whelan DB Bhandari M Systematic review of the treatment of periprosthetic distal femur fractures. J Orthop Trauma. (2014) 28(5):307–12. 10.1097/BOT.0000000000000002 24149447

[8] De Marco D Messina F Meschini C Oliva MS Rovere G Maccagnano G Periprosthetic knee fractures in an elderly population: open reduction and internal fixation vs distal femur megaprostheses. Orthop Rev (Pavia). (2022) 14(2):33772. 35774922 10.52965/001c.33772 PMC9239368

[9] Vertesich K Puchner SE Staats K Schreiner M Hipfl C Kubista B Distal femoral reconstruction following failed total knee arthroplasty is accompanied with risk for complication and reduced joint function. BMC Musculoskelet Disord. (2019) 20(1):47. 10.1186/s12891-019-2432-4 30704448 PMC6357401

[10] Wadhwa H Salazar BP Goodnough LH Van Rysselberghe NL DeBaun MR Wong HN Distal femur replacement versus open reduction and internal fixation for treatment of periprosthetic distal femur fractures: a systematic review and meta-analysis. J Orthop Trauma. (2022) 36(1):1–6. 10.1097/BOT.0000000000002141 34001801

